# Occurrence, Diversity, and Host Association of Intestinal *Campylobacter*, *Arcobacter*, and *Helicobacter* in Reptiles

**DOI:** 10.1371/journal.pone.0101599

**Published:** 2014-07-02

**Authors:** Maarten J. Gilbert, Marja Kik, Arjen J. Timmerman, Tim T. Severs, Johannes G. Kusters, Birgitta Duim, Jaap A. Wagenaar

**Affiliations:** 1 Department of Infectious Diseases and Immunology, Faculty of Veterinary Medicine, Utrecht University, Utrecht, The Netherlands; 2 Department of Pathobiology, Faculty of Veterinary Medicine, Utrecht University, Utrecht, The Netherlands; 3 Department of Medical Microbiology, University Medical Center Utrecht, Utrecht, The Netherlands; 4 WHO Collaborating Center for *Campylobacter*/OIE Reference Laboratory for Campylobacteriosis, Utrecht, The Netherlands; 5 Central Veterinary Institute of Wageningen UR, Lelystad, The Netherlands; GI Lab, United States of America

## Abstract

*Campylobacter*, *Arcobacter*, and *Helicobacter* species have been isolated from many vertebrate hosts, including birds, mammals, and reptiles. Multiple studies have focused on the prevalence of these *Epsilonproteobacteria* genera in avian and mammalian species. However, little focus has been given to the presence within reptiles, and their potential zoonotic and pathogenic roles. In this study, occurrence, diversity, and host association of intestinal *Epsilonproteobacteria* were determined for a large variety of reptiles. From 2011 to 2013, 444 cloacal swabs and fecal samples originating from 417 predominantly captive-held reptiles were screened for *Epsilonproteobacteria*. *Campylobacter*, *Arcobacter*, and *Helicobacter* genus specific PCRs were performed directly on all samples. All samples were also cultured on selective media and screened for the presence of *Epsilonproteobacteria*. Using a tiered approach of AFLP, *atpA*, and 16S rRNA sequencing, 432 *Epsilonproteobacteria* isolates were characterized at the species level. Based on PCR, *Campylobacter*, *Arcobacter*, and *Helicobacter* were detected in 69.3% of the reptiles; 82.5% of the chelonians, 63.8% of the lizards, and 58.0% of the snakes were positive for one or more of these genera. *Epsilonproteobacteria* were isolated from 22.1% of the reptiles and were isolated most frequently from chelonians (37.0%), followed by lizards (19.6%) and snakes (3.0%). The most commonly isolated taxa were *Arcobacter butzleri*, *Arcobacter skirrowii*, reptile-associated *Campylobacter fetus* subsp. *testudinum*, and a putative novel *Campylobacter* taxon. Furthermore, a clade of seven related putative novel *Helicobacter* taxa was isolated from lizards and chelonians. This study shows that reptiles carry various intestinal *Epsilonproteobacteria* taxa, including several putative novel taxa.

## Introduction

*Campylobacter*, *Arcobacter*, and *Helicobacter* species occupy a broad vertebrate host range and have been isolated from birds, mammals, and reptiles. These *Epsilonproteobacteria* genera are often found as commensals in the digestive tract, but some species are associated with disease in specific hosts [1]–[3]. The diverse genus *Arcobacter* also contains species free-living in the environment and species associated with non-vertebrate hosts [4]. *Campylobacter*, *Helicobacter*, and some *Arcobacter* species are mainly associated with endothermic vertebrate hosts, which is reflected in an optimal growth temperature of 30–42°C for most of the bacterial species [5]. However, *Campylobacter*, *Arcobacter*, and *Helicobacter* species have also been isolated from ectothermic reptiles [4], [6]–[10], which show a large variation in preferred body temperature and usually show considerable fluctuations in their body temperatures, often resulting in a lower average body temperature compared to most endothermic vertebrates [11]. A genetically distinct variant of *Campylobacter fetus* has been found in reptiles and humans that had direct or indirect contact with reptiles [6]–[13]. This reptile-associated *C*. *fetus* has been shown to cause infection in humans with underlying disease [12], [13]. Few bacterial species have been recognized as primary pathogens in reptiles [14], [15], and currently no cases have been reported of *Campylobacter* or *Arcobacter* species causing disease in reptiles, although a fatal septicemia in a pancake tortoise (*Malacochersus tornieri*) associated with an unidentified *Helicobacter* species has been described [9].

The role of reptiles in the epidemiology of *Epsilonproteobacteria* is currently unknown. Therefore, the aim of this study was to determine occurrence, diversity, and host association of intestinal *Epsilonproteobacteria* in reptiles.

## Materials and Methods

### Ethics statement

The procedures conducted on the reptiles in the study were merely combined with veterinary diagnostic and therapeutic acts and were therefore not considered to cause any additional discomfort. Consequently, the study was not considered an animal experiment under the Dutch Experiments on Animals Act (1977), making an assessment by an animal ethics committee unnecessary. The sampling was performed in strict accordance with the Dutch Law for Practicing Veterinary Medicine (1990). Fecal samples and cloacal swabs were collected as part of routine post mortem examination and diagnostic fecal examination for *Campylobacter* and other infectious agents. Sampling of live animals was performed by a veterinarian specialized in reptiles and amphibians (MK, Diplomate of the European College of Zoological Medicine, Herpetology). All owners provided consent for use of the samples collected from the reptiles.

### Sample collection and preparation

From 2011 to 2013, a total of 444 cloacal swabs (*n* = 363) and fecal samples (*n* = 81) were collected from 417 healthy, diseased, or dead reptiles (Table S1). Free-living (*n* = 2), recently wild-caught (*n* = 56), and captive-held animals (*n* = 359) were included. Sampled animals belonged to 97 unique species of 27 families (Table S1). Diversity was high amongst sampled animals and the reptilian orders Squamata (comprising suborders Lacertilia (lizards) and Serpentes (snakes)) and Testudines (chelonians) were covered. Samples were obtained from three zoos (*n* = 170), a veterinary pathology division (*n* = 77), a veterinary clinic specialized in reptiles and amphibians (*n* = 102), a veterinary microbiological diagnostic laboratory (*n* = 5), a pet shop specialized in reptiles and amphibians (*n* = 56), and private owners (*n* = 34), all located in the Netherlands. For 27 animals two samples per animal were included.

Cloacal swabs (Amies charcoal 114C and 116C; Copan, Brescia, Italy) were extracted in 500 µl phosphate buffered saline (PBS); fecal samples were diluted in PBS until a fluid suspension was obtained. Of the each suspension, 240 µl was used directly for culturing and 100 µl was stored at −80°C with 10 mM EDTA for DNA extraction and direct genus specific PCR analysis.

### Direct genus specific PCR analysis of samples

On all samples *Campylobacter*, *Arcobacter*, and *Helicobacter* genus specific PCRs were performed. For DNA isolation, 50 µl of lysis buffer (Cobas PCR Female Swab Sample Kit, Roche Molecular Systems Inc., Branchburg, USA) was added to 50 µl of each sample, followed by freezing at −80°C for 24 h and immediate heating at 95°C for 15 min in order to release the DNA and to inactivate DNAses. DNA was then extracted from this 100 µl sample on MagNA Pure96 (Roche), using the Roche MagNA Pure96 and Viral NA Small Volume Kit with the Viral NA Universal SV extraction protocol according to the manufacturer’s instructions. Final elution was in 100 µl, and 2 µl of these eluates was used as input for all PCRs. The *Arcobacter*, *Campylobacter*, and *Helicobacter* genus specific PCRs were performed as described previously [16]–[18], with slight adaptations. The *Arcobacter* genus specific PCR was performed using Go Taq Hot Start Green Master Mix (Promega, Madison, USA); total reaction volume was 25 µl and the PCR was run for 40 cycles on a 2720 Thermal Cycler (Applied Biosystems). Of each reaction, 10 µl was analyzed on a 1% agarose gel for the 1223 bp amplicon. Both *Campylobacter* and *Helicobacter* genus specific PCRs were performed using a LightCycler 480 II (Roche) and were run 45 cycles. LightCycler 480 Probes Master (Roche) was used for the *Campylobacter* genus specific PCR in a total reaction volume of 25 µl; LightCycler 480 SYBR Green I Master (Roche) was used for the *Helicobacter* genus specific PCR in a total reaction volume of 15 µl. Using the LightCycler 480 software (Roche), the *Campylobacter* and *Helicobacter* genus specific PCRs were analyzed based on absolute quantification and melting curves (range 85.5–87°C), respectively. *Arcobacter butzleri* strain SX-460, *Campylobacter fetus* strain NCTC 10842, and *Helicobacter pylori* strain J99 were used as reference strains. A subset of the amplicons was sequenced to validate the PCR results.

### Culturing

From each of the sample suspensions, 10 µl was plated on each of four different media: blood agar (Colombia agar with 5% sheep blood), CCD agar, Preston agar and Skirrow agar (Oxoid, Landsmeer, The Netherlands). Additionally, a membrane filtration method was used; 200 µl suspension was applied on a cellulose filter membrane (ME26; 50 mm; pore size 0.6 µm; Whatman, Dassel, Germany) placed on a blood agar plate, incubated at atmospheric conditions at 37°C for one hour, after which the filter membrane was discarded. All agar plates were incubated in a microaerobic atmosphere containing hydrogen (83.3% N_2_, 7.1% CO_2_, 3.6% H_2_, and 6% O_2_) at 37°C and screened for colonies showing *Epsilonproteobacteria*-like morphology after 24 h, 48 h, and after a week of incubation. Selected colonies were colorless, whitish, greyish, or brownish, often small, slow growing, and flat, with or without the tendency to spread and showing no or little hemolysis on blood containing media. The number and diversity of colonies showing *Epsilonproteobacteria*-like morphology varied considerably for each sample and determined the number of selected colonies per sample. On average 9.0 (±6.1) colonies per sample were subcultured onto a fresh blood agar plate, incubated at 37°C under microaerobic conditions until growth was visible, and Gram-stained. Gram-negative bacteria showing helical or curved rod-shaped microscopic morphology typical for most *Epsilonproteobacteria* were selected and stored at −80°C.

For molecular analysis of the isolates, bacterial genomic DNA of all isolates was extracted following the Gram-negative bacteria protocol of either the High pure PCR template preparation kit (Roche Diagnostics, Almere, The Netherlands) or Gentra Puregene yeast/bacteria kit (Qiagen, Venlo, The Netherlands).

### AFLP

Amplified fragment length polymorphism (AFLP) was used to analyze diversity amongst the isolates and for preliminary species identification by inclusion of well-characterized *Campylobacter*, *Arcobacter*, and *Helicobacter* strains. AFLP was performed on all isolates as described by Duim et al. [19], with 20 ng genomic DNA for each 14 µl restriction/ligation reaction mixture. Fingerprint data were created with a 3730 DNA analyzer (Applied Biosystems, Nieuwerkerk aan den IJssel, The Netherlands) and clustered (pairwise similarities; curve-based Pearson correlation; UPGMA clustering) using BioNumerics v.6.6 (Applied Maths, Sint-Martens-Latem, Belgium). For each sample one representative isolate per distinct fingerprint cluster (≥75% similarity) was selected for subsequent *atpA* and 16S rRNA sequencing.

### *atpA* and 16S rRNA sequencing for species identification of isolates

For further characterization and species identification of the isolates *atpA* sequencing was performed. The *atpA* (or *uncA*) locus, encoding the ATPase synthase α subunit, is part of the MLST-scheme for *Campylobacter* [20]. In *Epsilonproteobacteria*, *atpA* is conserved and has been used to improve *Campylobacteraceae* and *Helicobacteraceae* species identification by 16S rRNA sequencing [21], thereby making it a good candidate for (sub)species identification of the isolates. Two degenerate primer sets targeting *atpA* from a broad range of *Campylobacter*, *Arcobacter*, and *Helicobacter* species were used: atpAF/R [20] and HFatpF/R [22]. For isolates for which *atpA* could not be sequenced, 16S rRNA encoding DNA sequencing was used using the 16S rDNA targeting primers 27F and 1492R [23]. Each PCR amplification mixture contained 2.5 µM each primer, 1x Go Taq Hot Start Green Master Mix (Promega) or Hot Star Taq Master Mix (Qiagen) and 1 ng/µl genomic DNA. PCRs were performed on a 2720 Thermal Cycler (Applied Biosystems) with the following conditions: 30 s at 94°C, 30 s at 53°C, and 2 min at 72°C (35 (16S rRNA) or 40 cycles (*atpA*)). DNA sequencing was performed by Macrogen (Amsterdam, The Netherlands), and DNA sequences were assembled and analyzed using BioNumerics v.6.6 (Applied Maths). Species were identified by aligning and clustering DNA sequences (MegAlign; DNASTAR Lasergene v.10.1) with an extensive database containing most currently known *Epsilonproteobacteria* (*atpA*) or using NCBI BLASTn (16S rRNA). By combining AFLP and sequence data, the species or genus was identified for all isolates. In addition, for all isolated *Helicobacter* taxa and most described *Helicobacter* species, 16S rRNA sequences of one strain per taxon were aligned with Muscle and a 16S rRNA based dendrogram was created using the neighbor-joining method, with bootstrap values determined using 500 repetitions (MEGA v.5.2).

## Results

*Campylobacter*, *Arcobacter*, or *Helicobacter* were detected by PCR in 69.3% (289/417) of the reptiles; 82.5% (127/154) of Testudines, 63.8% (104/163) of Lacertilia, and 58.0% (58/100) of Serpentes were positive for one or more of these genera (Table 1). The occurrence of each *Epsilonproteobacteria* genus was highest in Testudines, followed by Lacertilia and Serpentes.

**Table 1:** Number of animals positive for each *Epsilonproteobacteria* taxon per reptilian (sub)order.

	Lacertilia (*n* = 163)		Serpentes (*n* = 100)		Testudines (*n* = 154)		Total (*n* = 417)	
	Animals (%)		Animals (%)		Animals (%)		Animals (%)	
	PCR	Culturing	PCR	Culturing	PCR	Culturing	PCR	Culturing
***Arcobacter*** ** genus**	23 (14.1)	6 (3.7)	13 (13.0)	2 (2.0)	47 (30.5)	25 (16.2)	83 (19.9)	33 (7.9)
*Arcobacter butzleri*		6 (3.7)		2 (2.0)		11 (7.1)		19 (4.6)
* Arcobacter cryaerophilus*		1 (0.6)		0 (0.0)		9 (5.8)		10 (2.4)
* Arcobacter skirrowii*		0 (0.0)		0 (0.0)		14 (9.1)		14 (3.4)
***Campylobacter*** ** genus**	62 (38.0)	18 (11.0)	32 (32.0)	3 (3.0)	93 (60.4)	39 (25.3)	187 (44.8)	60 (14.4)
* Campylobacter fetus* taxon 1*		9 (5.5)		3 (3.0)		11 (7.1)		23 (5.5)
* Campylobacter fetus* taxon 2*		0 (0.0)		0 (0.0)		1 (0.6)		1 (0.2)
* Campylobacter hyointestinalis*		0 (0.0)		0 (0.0)		9 (5.8)		9 (2.2)
* Campylobacter* taxon 1*		10 (6.1)		0 (0.0)		24 (15.6)		34 (8.2)
* Campylobacter* taxon 2*		0 (0.0)		0 (0.0)		1 (0.6)		1 (0.2)
***Helicobacter*** ** genus**	50 (30.7)	12 (7.4)	26 (26.0)	0 (0.0)	87 (56.5)	8 (5.2)	163 (39.1)	20 (4.8)
* Helicobacter* taxon 1*		1 (0.6)		0 (0.0)		0 (0.0)		1 (0.2)
* Helicobacter* taxon 2*		0 (0.0)		0 (0.0)		8 (5.2)		8 (1.9)
* Helicobacter* taxon 3*		4 (2.5)		0 (0.0)		0 (0.0)		4 (1.0)
* Helicobacter* taxon 4*		1 (0.6)		0 (0.0)		0 (0.0)		1 (0.2)
* Helicobacter* taxon 5*		1 (0.6)		0 (0.0)		0 (0.0)		1 (0.2)
* Helicobacter* taxon 6*		1 (0.6)		0 (0.0)		0 (0.0)		1 (0.2)
* Helicobacter* taxon 7*		4 (2.5)		0 (0.0)		0 (0.0)		4 (1.0)
* Sulfurospirillum* taxon*		0 (0.0)		0 (0.0)		1 (0.6)		1 (0.2)
**Total** **	**104 (63.8)**	**32 (19.6)**	**58 (58.0)**	**3 (3.0)**	**127 (82.5)**	**57 (37.0)**	**289 (69.3)**	**92 (22.1)**

*n*, total of sampled animals;

*, putative novel species or subspecies based on 16S rRNA sequence and AFLP fingerprint;

**, not sum of *Epsilonproteobacteria* positive animals in case of multiple *Epsilonproteobacteria* taxa per animal.

With an occurrence of 44.8% (187/417) in all animals, *Campylobacter* was detected with the highest frequency. Compared to *Campylobacter*, *Helicobacter* and *Arcobacter* were detected less frequently: 39.1% (163/417) and 19.9% (83/417), respectively.

Overall, 432 *Epsilonproteobacteria* isolates were obtained by culturing (Table S1). Of the 92 positive animals, on average 4.7 (±4.1) *Epsilonproteobacteria* isolates were obtained from each positive animal. Based on AFLP fingerprint data of all isolates, a selection of 188 isolates with unique patterns for each sample was made for species identification with *atpA* and/or 16S rRNA sequencing. Combining AFLP and sequence data, all isolates could be identified.

*Epsilonproteobacteria* belonging to the genera *Campylobacter*, *Arcobacter*, *Helicobacter*, and *Sulfurospirillum* were isolated from 22.1% (92/417) of the animals (Table 1). Examining the occurrence of *Epsilonproteobacteria* for each reptilian (sub)order included in this study, highest occurrence is observed in Testudines 37.0% (57/154), followed by Lacertilia 19.6% (32/163) and Serpentes 3.0% (3/100). Testudines showed the highest *Epsilonproteobacteria* diversity with up to four different taxa per animal. Amongst Testudines, terrestrial chelonians of the Testudinidae family showed the highest occurrence of *Epsilonproteobacteria*: while 49.4% (76/154) of the sampled chelonians and 18.2% (76/417) of the sampled reptiles belonged to the Testudinidae, these animals made up 80.4% (45/56) of the *Epsilonproteobacteria* positive chelonians (odds ratio 8.8; 95% confidence interval 4.0–19.4; *P*<0.0001) and 48.9% (45/92) of all *Epsilonproteobacteria* positive reptiles (odds ratio 9.1; 95% confidence interval 5.2–15.8; *P*<0.0001).

Detection rates in reptiles were lower based on culturing methods (22.1%) than based on genus specific PCRs (69.3%). Animals positive for *Epsilonproteobacteria* by culturing were all positive by genus specific PCR (*Campylobacter* and *Helicobacter*), except one (*Arcobacter*; 97.0% (32/33)).

The majority of isolated *Epsilonproteobacteria* belonged to *Campylobacter* taxa (Table 1). Members of the *Campylobacter* genus were isolated from 14.4% (60/417) of the reptiles. Based on AFLP, *atpA*, and 16S rRNA sequence similarity, *Campylobacter* taxa genetically distinct from described species were identified, likely representing novel species and subspecies. *Campylobacter* taxon 1 and *C*. *fetus* taxon 1 were the most frequently isolated *Epsilonproteobacteria* taxa. Next to these taxa, *C*. *hyointestinalis*, *Campylobacter* taxon 2, and *C*. *fetus* taxon 2 were isolated from Testudines. The latter was distinct from other *C*. *fetus* taxa and all isolates were obtained from one animal (*Chelonoidis carbonaria*). *Campylobacter* taxon 1 was isolated from Testudines 15.6% (24/154) and Lacertilia 6.1% (10/163). Occurrence of *C*. *fetus* taxon 1 was highest in Testudines 7.1% (11/154), followed by Lacertilia 5.5% (9/163) and Serpentes 3.0% (3/100).

*Arcobacter* species were isolated from 7.9% (33/417) of the reptiles, predominantly from Testudines (Table 1). Species isolated were *Arcobacter butzleri*, *Arcobacter cryaerophilus*, and *Arcobacter skirrowii*. *A*. *butzleri* was isolated from Lacertilia, Serpentes, and Testudines while *A*. *skirrowii* was exclusively isolated from Testudines.

**Figure 1:**
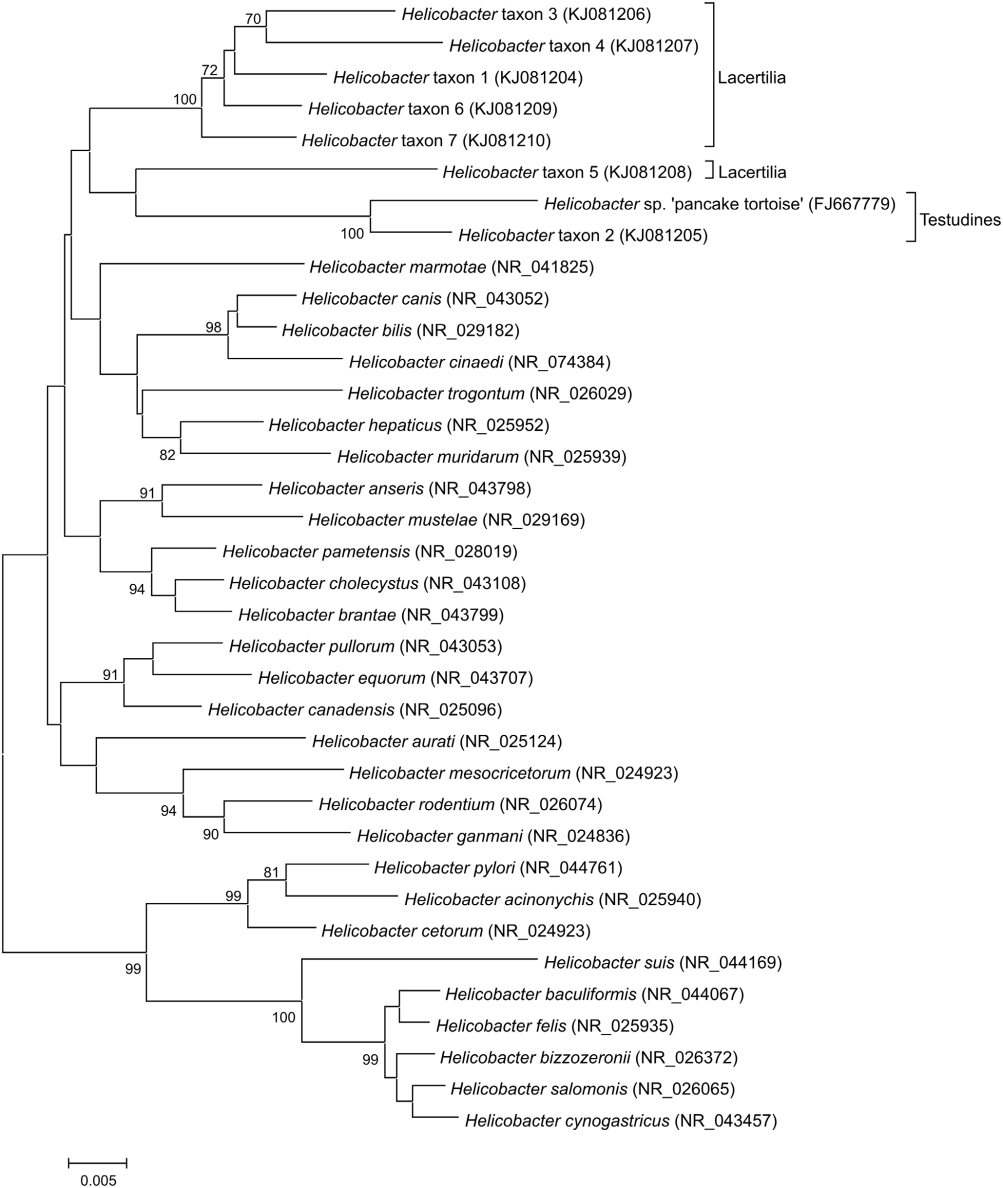
Neighbor-joining dendrogram based on 16S rRNA showing the phylogenetic position of all isolated *Helicobacter* taxa. Bootstrap values (≥70%) based on 500 repetitions are shown at the nodes of the dendrogram. The origins of the isolated *Helicobacter* taxa are indicated: Lacertilia (lizards) or Testudines (chelonians).

Compared to *Arcobacter* and *Campylobacter*, *Helicobacter* occurrence was low, 4.8% (20/417), but diversity was high. All isolates belonged to seven unknown taxa and were obtained from Lacertilia and Testudines (Table 1). A 16S rRNA based neighbor-joining dendrogram shows that these seven taxa cluster together, but apart from other *Helicobacter* species isolated from mammals and birds (Figure 1). Within this cluster, three distinct clusters are separated by deep branching. Interestingly, these clusters suggest *Helicobacter* host association: five out of six taxa obtained from Lacertilia clustered together, whereas *Helicobacter* taxon 2, obtained from Testudines, clustered with a previously described *Helicobacter* species isolated from a pancake tortoise (*Malacochersus tornieri*) [9].

In addition to *Campylobacter*, *Arcobacter*, and *Helicobacter* taxa, isolates of an unknown *Sulfurospirillum* taxon were obtained from a Malaysian giant turtle (*Orlitia borneensis*) (Table 1).

For each putative novel taxon, 16S rRNA sequences have been deposited in GenBank and accession numbers have been provided (Table S2).

The detection rates for each culturing method based on sampled animals were: filter method 85.9% (79/92), CCD 48.9% (45/92), Preston 46.7% (43/92), Skirrow 40.2% (37/92), and blood agar 13.0% (12/92) (Table 2). All frequently occurring *Campylobacter* and *Arcobacter* taxa (≥1 positive animal for each taxon) could be isolated using the filter method, CCD, Preston, and Skirrow agar. Although some *Helicobacter* isolates were obtained from Preston and Skirrow agar, the majority (53/59) was isolated from blood agar or by the filter method. The *Sulfurospirillum* isolates were obtained by the filter method and from Preston agar.

**Table 2:** Number of animals positive for each *Epsilonproteobacteria* taxon per culture medium used.

	Blood agar	Filter	CCD	Preston	Skirrow	Total
Taxon	Animals (%)	Animals (%)	Animals (%)	Animals (%)	Animals (%)	Animals (%)
*Arcobacter butzleri*	0 (0.0)	12 (63.2)	7 (36.8)	4 (21.1)	9 (47.4)	**19 (20.2)**
*Arcobacter cryaerophilus*	0 (0.0)	9 (90.0)	4 (40.0)	5 (50.0)	4 (40.0)	**10 (10.9)**
*Arcobacter skirrowii*	1 (7.1)	13 (92.9)	7 (50.0)	3 (21.4)	3 (21.4)	**14 (15.2)**
*Campylobacter fetus* taxon 1*	0 (0.0)	14 (60.9)	16 (69.6)	8 (34.8)	10 (43.5)	**23 (25.0)**
*Campylobacter fetus* taxon 2*	0 (0.0)	0 (0.0)	1 (100.0)	1 (100.0)	0 (0.0)	**1 (1.1)**
*Campylobacter hyointestinalis*	2 (22.2)	3 (33.3)	6 (66.7)	6 (66.7)	2 (22.2)	**9 (9.8)**
*Campylobacter* taxon 1*	3 (8.8)	18 (52.9)	9 (26.5)	18 (52.9)	8 (23.5)	**34 (37.0)**
*Campylobacter* taxon 2*	0 (0.0)	0 (0.0)	0 (0.0)	0 (0.0)	1 (100.0)	**1 (1.1)**
*Helicobacter* taxon 1*	0 (0.0)	1 (100.0)	0 (0.0)	0 (0.0)	0 (0.0)	**1 (1.1)**
*Helicobacter* taxon 2*	1 (12.5)	8 (100.0)	0 (0.0)	0 (0.0)	1 (12.5)	**8 (8.7)**
*Helicobacter* taxon 3*	2 (50.0)	4 (100.0)	0 (0.0)	1 (25.0)	1 (25.0)	**4 (4.3)**
*Helicobacter* taxon 4*	0 (0.0)	1 (100.0)	0 (0.0)	0 (0.0)	0 (0.0)	**1 (1.1)**
*Helicobacter* taxon 5*	0 (0.0)	1 (100.0)	0 (0.0)	0 (0.0)	0 (0.0)	**1 (1.1)**
*Helicobacter* taxon 6*	0 (0.0)	1 (100.0)	0 (0.0)	0 (0.0)	0 (0.0)	**1 (1.1)**
*Helicobacter* taxon 7*	3 (75.0)	4 (100.0)	0 (0.0)	0 (0.0)	2 (50.0)	**4 (4.3)**
*Sulfurospirillum* taxon*	0 (0.0)	1 (100.0)	0 (0.0)	1 (100.0)	0 (0.0)	**1 (1.1)**
**Total** **	**12 (13.0)**	**79 (85.9)**	**45 (48.9)**	**43 (46.7)**	**37 (40.2)**	**92 (100.0)**

*, putative novel species or subspecies based on 16S rRNA sequence and AFLP fingerprint;

**, not sum of *Epsilonproteobacteria* positive animals in case of multiple *Epsilonproteobacteria* taxa per animal.

## Discussion

*Epsilonproteobacteria* were present in all reptilian orders examined. Chelonians showed highest *Epsilonproteobacteria* occurrence and diversity. Based on direct genus specific PCR analysis, *Campylobacter* occurrence was highest in all reptilian (sub)orders, followed by *Helicobacter* and *Arcobacter*. The majority of isolated *Epsilonproteobacteria* belonged to the genera *Campylobacter* and *Arcobacter*. *Helicobacter* was isolated less frequently. One undescribed *Sulfurospirillum* taxon was isolated. All currently described *Sulfurospirillum* species were isolated from environmental sources, potentially making this the first *Sulfurospirillum* taxon isolated from an animal host. Most commonly isolated taxa were *Campylobacter* taxon 1 and *Campylobacter fetus* taxon 1. *Campylobacter* taxon 1 has been proposed as a novel species, *Campylobacter* “*iguaniorum*” (Cig) (unpublished data). The 16S rRNA sequences of Cig were homologous to a recently described *Campylobacter* strain isolated from a leopard tortoise (*Stigmochelys pardalis*) [10]. *Campylobacter fetus* taxon 1 has recently been described as a novel subspecies, *Campylobacter fetus* subsp. *testudinum* (Cft) [24]. Cft is homologous to the previously described reptile-associated *C*. *fetus* [6]–[8], [12], [13] and similar detection rates in reptiles as found in this study have been reported [8]. A demarcated *Campylobacter* species distribution was observed in this study: species commonly found in mammals and birds, such as *C*. *jejuni*, *C*. *coli*, and *C*. *lari*, were not found in reptiles. The reverse was observed for Cig and Cft, which were isolated from reptiles, but have not (Cig) or rarely (Cft) been isolated from endothermic vertebrates. This differential distribution of *Campylobacter* species likely reflects the body temperature of the host, which is fluctuating and on average lower in ectothermic reptiles (with minimum and maximum voluntary temperature ranging from 5–46°C and a mean voluntary temperature ranging from 20–35°C for most families) than in endothermic birds and mammals [11]. Cft and Cig grow well at lower temperatures (20–37°C) and show less or no growth at higher temperatures (42°C), which might be an adaptation to their reptilian hosts. Many *Epsilonproteobacteria* taxa did not show a strict host association within the examined reptiles, i.e. the same taxon could be isolated from Lacertilia, Serpentes, and Testudines. Especially some of the *Arcobacter* and *Campylobacter* taxa displayed broad host ranges. In contrast, the isolated *Helicobacter* taxa suggest a more confined host association, as based on 16S rRNA phylogeny the taxa were separated in a cluster with isolates originating from lizards and a cluster with isolates originating from chelonians of the Testudinidae family. All *Helicobacter* taxa isolated from reptiles clustered separate from *Helicobacter* species isolated from mammals and birds, and, based on 16S rRNA divergence, likely represent novel species.

Interestingly, all snakes carrying Cft were sick or deceased, while most lizards and chelonians carrying Cft appeared clinically healthy. Of all isolates obtained from snakes, 88.0% (22/25) were Cft, and no other *Epsilonproteobacteria* taxa except three *A*. *butzleri* isolates were obtained from these animals. Necropsy showed that all Cft infected snakes had severe infections in the colon or abdominal cavity. It is tempting to speculate that Cft is associated with disease, but due to the low number of animals this should be interpreted with care. Little is known about pathogenicity in reptiles of other *Epsilonproteobacteria* taxa, although a fatal septicemia in a pancake tortoise (*Malacochersus tornieri*), associated with a *Helicobacter* species related to *Helicobacter* taxon 2, isolated from spur-thighed tortoises (*Testudo graeca*) and Russian tortoises (*Agrionemys horsfieldii*), has been described [9]. The majority of *Epsilonproteobacteria* species isolated from reptiles during this study (*C*. *fetus*, *C*. *hyointestinalis*, *A*. *butzleri*, *A*. *cryaerophilus*, and *A*. *skirrowii*) have been shown to be potentially pathogenic in humans [1], [25], [26]. Although for reptilian *C*. *fetus* there appears to be an association between contact with reptiles and infection in humans [6], [12], [13], to this date, no such association has been described for the other *Epsilonproteobacteria* species. The growing number of reptiles kept as pets in some European countries [27] and increased farming of reptiles, predominantly freshwater turtles for human consumption, in Asian countries [28], might increase the risk of reptilian derived *Epsilonproteobacteria* infections in humans.

It has to be noted that most samples were obtained from captive-held reptiles, which may lead to other transmission dynamics and an intestinal microbiota composition distinct from free-living reptiles, as has been shown for other vertebrates [29]. Indeed, AFLP indicated that unrelated reptile species having direct or indirect contact (same housing, same zoo, or same shipment) can harbor the same specific *Epsilonproteobacteria* variants, suggesting horizontal transmission (data not shown). Fecal-oral transmission is likely, especially in captive animals and species displaying coprophagy, although a common feed related origin cannot be excluded. Particularly chelonians of the Testudinidae family commonly display coprophagy, presumably to supplement their intestinal microbiota and optimize digestion of their herbivorous diet. This behavior could partly explain the frequent occurrence and high diversity of intestinal *Epsilonproteobacteria* in these chelonians. Nevertheless, the majority of *Epsilonproteobacteria* species were also isolated from recently caught free-living reptiles. Studies comparing *Salmonella* carriage in captive-held and free-living reptiles showed that occurrence was lower in the latter [30], [31], but additional sampling of free-living reptiles would be necessary to elucidate the native *Epsilonproteobacteria* carrier status. In addition to this, analysis of additional samples obtained from species of the excluded orders Rhynchocephalia and Crocodilia is required to obtain a more precise estimation of the intestinal *Epsilonproteobacteria* occurrence in reptiles.

The methods used in this study might have influenced the observed intestinal *Epsilonproteobacteria* occurrence, as described below. A total of 69.1% (PCR) and 22.3% (culturing) of the cloacal swabs, and 55.6% (PCR) and 16.0% (culturing) of the fecal samples was positive for *Epsilonproteobacteria*. Although there was no statistical difference between the detection rates for cloacal swabs and fecal samples (two tailed Fisher exact test; *P* = 0.03 (PCR) and *P* = 0.23 (culturing)), it is uncertain whether these detection rate differences are inherent to the sample type or merely a result of stochastic processes. Of 27 animals two samples were included, which potentially increased the detection rate: 12 (PCR) and 22 (culturing) cases contained two negative samples; eight (PCR) and three (culturing) cases contained one positive sample; seven (PCR) and two (culturing) cases contained two positive samples. The culturing temperature was 37°C, which may have been too high for culturing potential *Campylobacter*, *Arcobacter*, and *Helicobacter* from reptiles with low preferred body temperatures. A subset of samples was cultured at both 30°C and 37°C to determine the effect of temperature on growth rate and species composition. Although a lower growth rate was observed at 30°C, colony composition appeared similar at both temperatures (data not shown). The culture media were selected to promote *Epsilonproteobacteria* growth and inhibit growth of non-*Epsilonproteobacteria*. Although most of the antimicrobial containing media used are optimized for *C*. *jejuni* and *C*. *coli*, the results of the filter method showed high agreement with media containing antimicrobials for all isolated *Campylobacter* and *Arcobacter* taxa (positive animals >1). Most *Helicobacter* taxa were isolated using media without antimicrobials. The discrepancy in detection rates between culturing and direct genus specific PCR was most prominent in the *Helicobacter* genus, suggesting that the culturing methods were suboptimal for *Helicobacter*. Also, non-motile *Epsilonproteobacteria* species, such as *C*. *gracilis*, will likely not pass through the filter. Microaerobic conditions are preferred by most culturable *Epsilonproteobacteria* species [5], although some species, such as *C*. *gracilis* and *C*. *rectus*, prefer anaerobic conditions. The presence of hydrogen, formate, or succinate as an electron source is essential for growth of some *Campylobacter* species. Furthermore, only 10 µl (≤2%) of the fecal suspension was plated on selective media, and a selection of colonies displaying *Epsilonproteobacteria*-like morphology was isolated. Since not all *Epsilonproteobacteria* display the typical helical or curved rod shape, screening microscopically for this feature might lead to an underestimation of the occurrence.

As expected, direct genus specific PCRs resulted in higher detection rates compared to culturing. Nearly all samples positive for *Epsilonproteobacteria* by culturing were positive by PCR as well. Members of the *Campylobacter* genus were most frequently detected by both PCR and culturing. Remarkably, members of the *Arcobacter* genus were detected least frequently by PCR, whereas members of the *Helicobacter* genus were detected least frequently by culturing. This discrepancy indicates that the *Arcobacter* and *Helicobacter* PCRs likely have different sensitivities and that both genera show differential growth on the media used. Indeed, many members of the *Helicobacter* genus are considered fastidious organisms [2]. Since *Epsilonproteobacteria* were detected on genus level by PCR and higher detection rates were observed by PCR than by culturing, fastidious or non-culturable species might not have been identified, which could have led to an underestimation of the true *Epsilonproteobacteria* diversity. Ideally, one PCR detecting all *Epsilonproteobacteria* directly in crude samples would overcome the potential differences in sensitivities between PCRs. Although a PCR-RFLP method targeting 16S rRNA of most *Campylobacter*, *Arcobacter*, and *Helicobacter* species has been described [32], this assay is designed for species identification of isolates, and not for direct assessment of samples. While molecular methods are useful, isolates obtained by culturing are preferred for further analysis, such as examination of epidemiology, diversity, and phenotypic characters, making both methods complementary.

The polyphasic approach based on direct genus specific PCR and culturing was useful to determine intestinal *Epsilonproteobacteria* occurrence and diversity from genus to subspecies level. Our results show that reptiles comprise a significant reservoir for *Epsilonproteobacteria*. A clade of related putative novel *Helicobacter* taxa appears to be specifically associated with reptiles. *Arcobacter butzleri*, *Arcobacter skirrowii*, *Campylobacter fetus* subsp. *testudinum*, and *Campylobacter* “*iguaniorum*” represent a major portion of the *Epsilonproteobacteria* isolated from reptiles, chelonians in particular. The *Campylobacter* taxa are thus far predominantly found in reptiles, which may constitute their primary reservoir.

## Supporting Information

Table S1 **Number of samples, animals, and detected** ***Epsilonproteobacteria*** **for all reptilian species included in this study.** Epsilon, *Epsilonproteobacteria*; A, *Arcobacter* genus; Ab, *Arcobacter butzleri*; Ac, *Arcobacter cryaerophilus*; As, *Arcobacter skirrowii*; C, *Campylobacter* genus; Cf1, *Campylobacter fetus* taxon 1; Cf2, *Campylobacter fetus* taxon 2; Ch, *Campylobacter hyointestinalis*; C1, *Campylobacter* taxon 1; C2, *Campylobacter* taxon 2; H, *Helicobacter* genus; H1, *Helicobacter* taxon 1; H2, *Helicobacter* taxon 2; H3, *Helicobacter* taxon 3; H4, *Helicobacter* taxon 4; H5, *Helicobacter* taxon 5; H6, *Helicobacter* taxon 6; H7, *Helicobacter* taxon 7; S, *Sulfurospirillum* taxon.
(XLSX)

Table S2 **GenBank accession numbers of the 16S rRNA sequences for all putative novel** ***Epsilonproteobacteria*** **taxa isolated during this study.** *, 16S rRNA sequence deposited at GenBank prior to this study.
(XLSX)
